# Identification of AKIRIN2 as a potential biomarker and correlation with immunotherapy in gastric adenocarcinoma by integrated bioinformatics analysis

**DOI:** 10.1038/s41598-022-12531-8

**Published:** 2022-05-19

**Authors:** Shaopeng Sun, Jiajia Chen, Chunyan Weng, Yifan Lu, Chang Cai, Bin Lv

**Affiliations:** 1 The First Clinical Medical College of Zhejiang Chinese Medical University, Hangzhou, China; 2 Department of Anesthesiology, The First Affiliated Hospital of Zhejiang Chinese Medical University (Zhejiang Provincial Hospital of Traditional Chinese Medicine), Hangzhou, China; 3 Department of Gastroenterology, The First Affiliated Hospital of Zhejiang Chinese Medical University (Zhejiang Provincial Hospital of Traditional Chinese Medicine), Hangzhou, China

**Keywords:** Cancer, Immunology

## Abstract

Gastric adenocarcinoma is major type of gastric cancer that endangers human health. AKIRIN2 has been shown to be associated with cholangiocarcinoma promoting invasion and angiogenesis. In this study, AKIRIN2 is highly expressed in Gastric adenocarcinoma through bioinformatics analysis based on Stomach adenocarcinoma samples data from The Cancer Genome Atlas. Correlation analysis showed that the high-expression of AKIRIN2 was associated with poor survival rate compared to the low-expression group. Univariate and multivariate Cox regression analyses determined the correlation between clinical characteristics and overall survival. Next, the correlation between AKIRIN2 and immune infiltration was evaluated. The distribution of 24 immune cells and their correlation with the expression of AKIRIN2 were explored using the immune cell database. In addition, three Immune cell methods were used to verify the positive correlation between immune cells and AKIRIN2. Also, Genomics of Drug Sensitivity in Cancer database was utilized to verify the correlation between AKIRIN2 expression level and the efficacy of chemotherapy and immunotherapy. The results showed that AKIRIN2 is an effective biomarker of Gastric adenocarcinoma prognosis, which can guide chemotherapy and immunotherapy and clarify the progress of Gastric adenocarcinoma promoted by immune microenvironment.

## Introduction

Due to the lack of effective biomarkers, the prognosis of gastric cancer (GC) is a critical issue^1^. Gastric adenocarcinoma (GA) is a major type of GC and is mainly detected in the advanced stage. It ranked fifth in cancer incidence rate and third in death rate^2^. The early stage of GC is usually asymptomatic, and most patients who visit the hospital due to the symptoms are often diagnosed with advanced GC, which is a common reason for the high mortality of GC^3^. Although the survival rate of early gastric cancer is high, patients often miss the optimal operation time due to the low rate of early diagnosis^4^. Therefore, finding new and effective biomarkers to improve clinical efficacy is a research hotspot.

AKIRIN consists of a pair of highly evolved conserved nuclear factors. Two isoforms are detected in human beings, namely AKIRIN1 and AKIRIN2^5^. AKIRIN2 has been proved to be a key factor in embryonic development and innate immune response^6^ and confirmed to be upregulated in human primary glioblastoma^7^. A recent study of cholangiocarcinoma has shown that AKIRIN2 is regulated by the upstream mir-490-3p at post transcription level, while the downstream participates in the epithelial mesenchymal transformation process through IL-6/STAT3/VEGFA signal transduction, which plays a role in tumor growth and metastasis^8^. However, the role of AKIRIN2 in human diseases, especially cancer, is still poorly studied.

A compelling body of evidence has demonstrated that GA has a specific tumor microenvironment (TME), a signature suitable to promote tumor progression and metastasis^9^. TME consists of complex components, such as immune cells, fibroblasts, lymphocytes, blood vessels, and extracellular matrix^10^. Several studies have confirmed that tumor-infiltrating immune cells play a leading role in TEM^11^. The changes in TME can reduce the ability of tumor autoimmunity to monitor and eliminate tumor cells. Guo et.al established a multiscale model, which revealed the competition between inflammation and metabolism-immune and further explained the role of the imbalance of immune microenvironment in triggering the transformation of inflammation and cancer^12^. However, the role of immune cells in TME during tumor development remains largely unknown, especially in GA.

Immunotherapy improves the curative effect of patients with gastric tumor^13^. Compared to traditional therapies, immunotherapy, such as those targeting programmed death-ligand 1 (PD-L1), programmed death 1 (PD1), and cytotoxic T lymphocyte-associated protein 4 (CTLA4), have shown significant advantages in survival^14^. Although significant progress has been made in advanced GC, it should be noted that PD-1/PD-L1 inhibitors are not suitable for all patients with advanced GC, and immunobiological markers could screen suitable patients for PD-1/PD-L1 inhibitors.

In this study, we analyzed the transcriptome and clinical data of 277 GA patients from The Cancer Genome Atlas (TCGA) to identify the correlation between AKIRIN2 and clinical information. The expression level of AKIRIN2 was verified by the GENE EXPRESSION OMNIBUS (GEO) database. Moreover, we used ImmuCellAI, CIBERSORT, and TIMER to analyze the immune system infiltration data of GA in the TCGA database. Based on the genomics of drug sensitivity in cancer (GDSC) database, the response of patients with high- and low-expression of AKIRIN2 to chemotherapy drugs was explored. In addition, a specific cohort was utilized to confirm that high-expression of AKIRIN2 showed a satisfactory response to PD-1 inhibitor immunotherapy.

## Materials and methods

### Patients and samples

Stomach adenocarcinoma (STAD) patients from TCGA were included in this study. The gene expression profiling and clinical information were downloaded from UCSC Xena (http://xena.ucsc.edu), an online tool for public and private multi-omics and clinical/phenotype data^15^. After matching the clinical information, the patients who lack clinical information were excluded; finally, 32 cases of adjacent normal tissues and 277 cases of GA were included in this study. We also downloaded the GSE19826 dataset from the GEO database (https://www.ncbi.nlm.nih.gov/geo/), including 15 cases of adjacent normal tissues and 12 cases of GA tissues. T-test was used to evaluate the statistical difference between the tumor and normal gene expression.

### Assessment of prognostic clinical characteristics and AKIRIN2 in GA cohort

According to the expression level of AKIRIN2, we divided 277 GA samples into high- and low-expression groups. The correlation between AKIRIN2 expression and clinical features was analyzed by chi-square test. Kaplan–Meier (K–M) survival curve and log-rank test were used to analyze the difference in the prognosis of patients between the high- and low-expression groups. Next, we performed univariate and multivariate Cox proportional hazards regression analysis on the prognostic effect of AKIRIN2. P-value < 0.05 indicated statistical significance. K–M plot and forest plot were generated by ‘ggplot2’^16^ and ‘forest’ R packages in R software (version4.0, https://www.r-project.org).

### Functional and pathway enrichment analysis

We used the R package "limma"^17^ to analyze the differentially expressed genes (DEGs) between the high- and low-expression groups of AKIRIN2 and screened the DEGs with log2 (fold-change) > 0.3, P < 0.05 for pathway enrichment analysis. Gene Ontology (GO) analysis is used to describe the role of genes and proteins in cells to comprehensively describe the properties of genes and gene products in the organism data^18^. Three categories in GO database were biological process (BP), cellular component (CC), and molecular function (MF). Each category describes the molecular functions of the gene products, their cellular environment, and the related biological processes. Kyoto Encyclopedia of Genes and Genomes (KEGG) systematically analyzes the functions of genes based on the known biological processes in cells and the optimal interpretation of gene functions^19–22^. Hallmark gene sets^23^ are coherently expressed signatures derived by aggregating many Molecular Signatures Database to represent defined biological states or processes. In the current study, we analyzed the DEGs based on the GO function, KEGG, and Hallmark pathway enrichment analysis.

All the figures were generated by the R software (version4.0, https://www.r-project.org). The R-function “enrichGo” is used to construct the GO analysis annotation chart. The R function “enrichKEGG” and “cnetplot” was utilized to draw the KEGG pathway, and “cluster profiler” was used to display the Hallmark pathway. The adjusted P-value < 0.05 was set as the cutoff threshold of the displayed pathway.

### Correlation between AKIRIN2 and immune infiltration in GA

The composition and abundance of immune cells in TME influence the tumor progression and prognosis^24^. ImmuCellAI can estimate the abundance of 24 types of immune cells from gene expression data sets, among which 18 were T cell subtypes and six other immune cells^25^. ImmuCellAI detected the abundance of immune cells, and the differences in the infiltration immune cells of 277 GA were analyzed. Moreover, CIBERSORT^26^ and TIMER^27^ methods were used to evaluate the results of tumor immune infiltration analysis of the ImmuCellAI methods. The figure was generated by Microsoft Office Excel (version 2020, https://www.microsoft.com).

### Prediction for chemo/immunotherapeutic response

GDSC^28^ is the largest database used to evaluate drug sensitivity and molecular markers of drug response in tumor cells. Hence, we predicted the chemotherapeutic response for each sample based on GDSC. The prediction was based on the R package “pRRophetic”^29^. Three commonly used chemical drugs were selected: cisplatin, paclitaxel, and 5-fluorouracil. We estimated the half-maximal inhibitory concentration (IC50) of the samples by ridge regression and compared the responses of patients with high and low expression of AKIRIN2 to the above three drugs.

Tumor immune dysfunction and exclusion (TIDE) algorithms^30^ and subclass mapping^31^ were used to predict the clinical response to immune checkpoints between high- and low-expression groups. We collected the Anderson melanoma cohort^32^ treated with CTLA-4 or PD-1 blockade therapy to verify whether the expression of AKIRIN2 is related to the response of immunotherapy. The violin plot and submap plot were generated with the ‘ggplot2’ and ‘pheatmap’ R package respectively in R software (version4.0, https://www.r-project.org).

### IHC

Ten paraffin samples of gastric cancer and corresponding normal adjacent tissues were chosen randomly for immunohistochemistry (IHC). The protein levels of AKIRIN2 (1:500 SAB) in formalin-fixed paraffin embedded gastric cancer tissues and adjacent non-tumor tissues were detected by IHC. First, the sections were incubated with AKIRIN2 antibody (HRP Conjugated) and then counterstained with Hematoxylin. Finally, two professional pathologists will score respectively, and the score with different opinions will be determined through discussion. The semi-quantitative method is based on the intensity of staining (0, negative; 1, weak; 2, moderate; 3, strong) and the percentage of positively staining (0, no staining; 1, 1–24%; 2, 25–49%; 3, 50–74%; 4, 75–100%). The final score of immunostaining is calculated by multiplying the staining intensity to the positive staining percentage^33^.

### Statistical analysis

Statistical analyses were carried out using the R software (version 4.0). Student’s t-test was used to analyze differences in gene expression between the tumor and normal groups. The association between clinical data and AKIRIN2 expression was assessed using the chi-square test. The prognostic analysis of GA patients was evaluated using the K–M method and the univariate and multivariate Cox regression analysis. P-value < 0.05 indicated statistical significance.

### Ethics approval

The research protocol for the study was approved by the Ethics Committee of The First Affiliated Hospital, Zhejiang Chinese Medicine University (number 2021-KL-142-01). All experiments were performed in accordance with relevant guidelines and regulations.

## Results

### AKIRIN2 has high expression in GA

**Figure 1:**
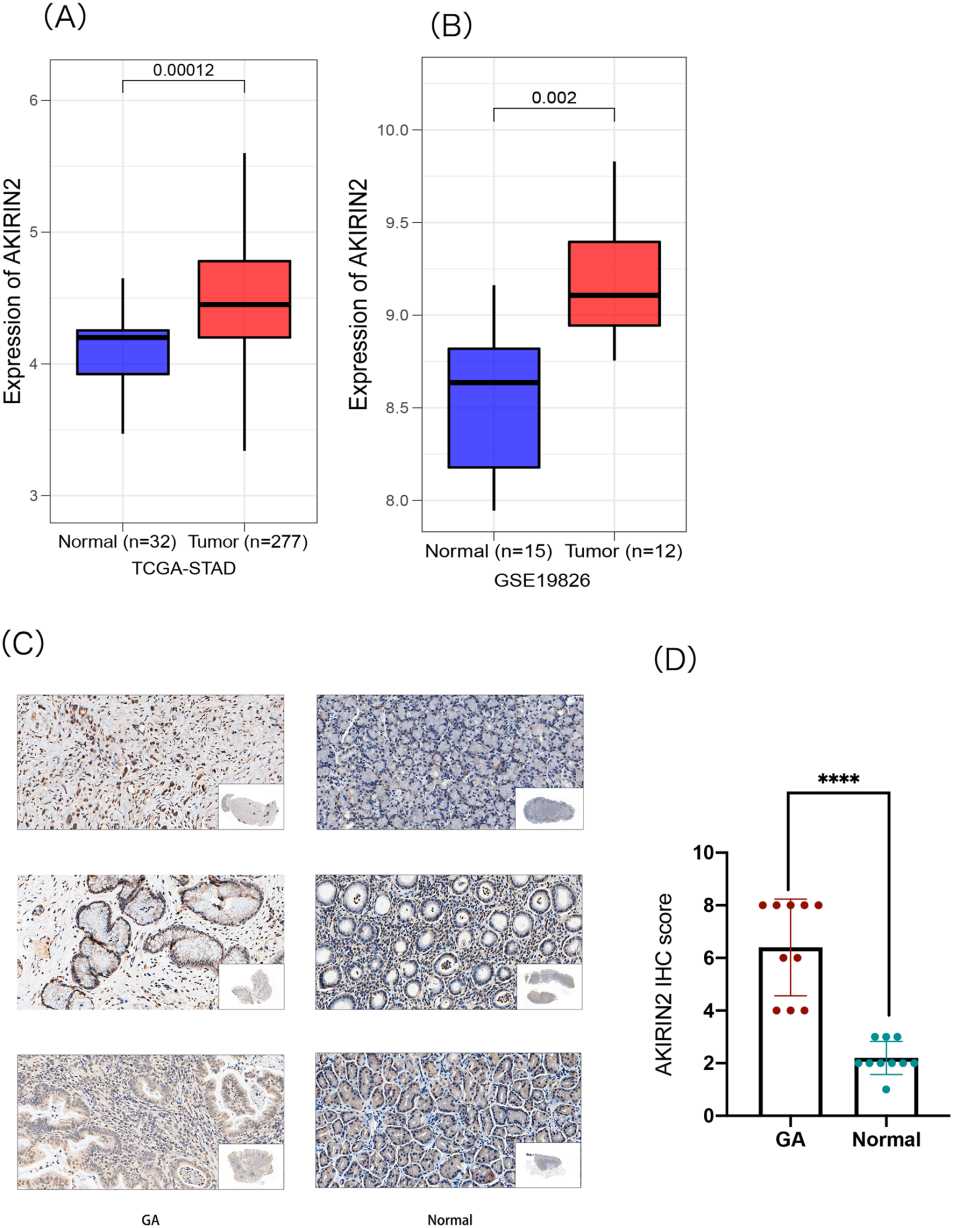
Distribution of AKIRIN2 between GA and normal tissues. (**A**) Expression of AKIRIN2 based on TCGA-STAD (P value = 0.00012). (**B**) Expression of AKIRIN2 based on GSE19826 (P value = 0.002). (**C**,**D**) IHC analysis of GA and normal tissues. ****P < 0.0001.

Based on the TCGA database, we explored the expression of AKIRIN2 in 227 GA samples and 32 normal samples. The findings reflected that AKIRIN2 is highly expressed in tumor samples (Fig. 1A). To confirm this result, we used GSE19826 (12 tumor samples and 15 normal samples) from GEO database to verify (Fig. 1B). The analysis results of the two databases showed that AKIRIN2 is highly expressed in tumors. To further determine the results, we randomly selected 10 gastric cancer samples and corresponding normal adjacent from the sample library. We found that AKIRIN2 was up-regulated in gastric cancer (Fig. 1C,D).

### Prognostic value of AKIRIN2 in GA

**Figure 2:**
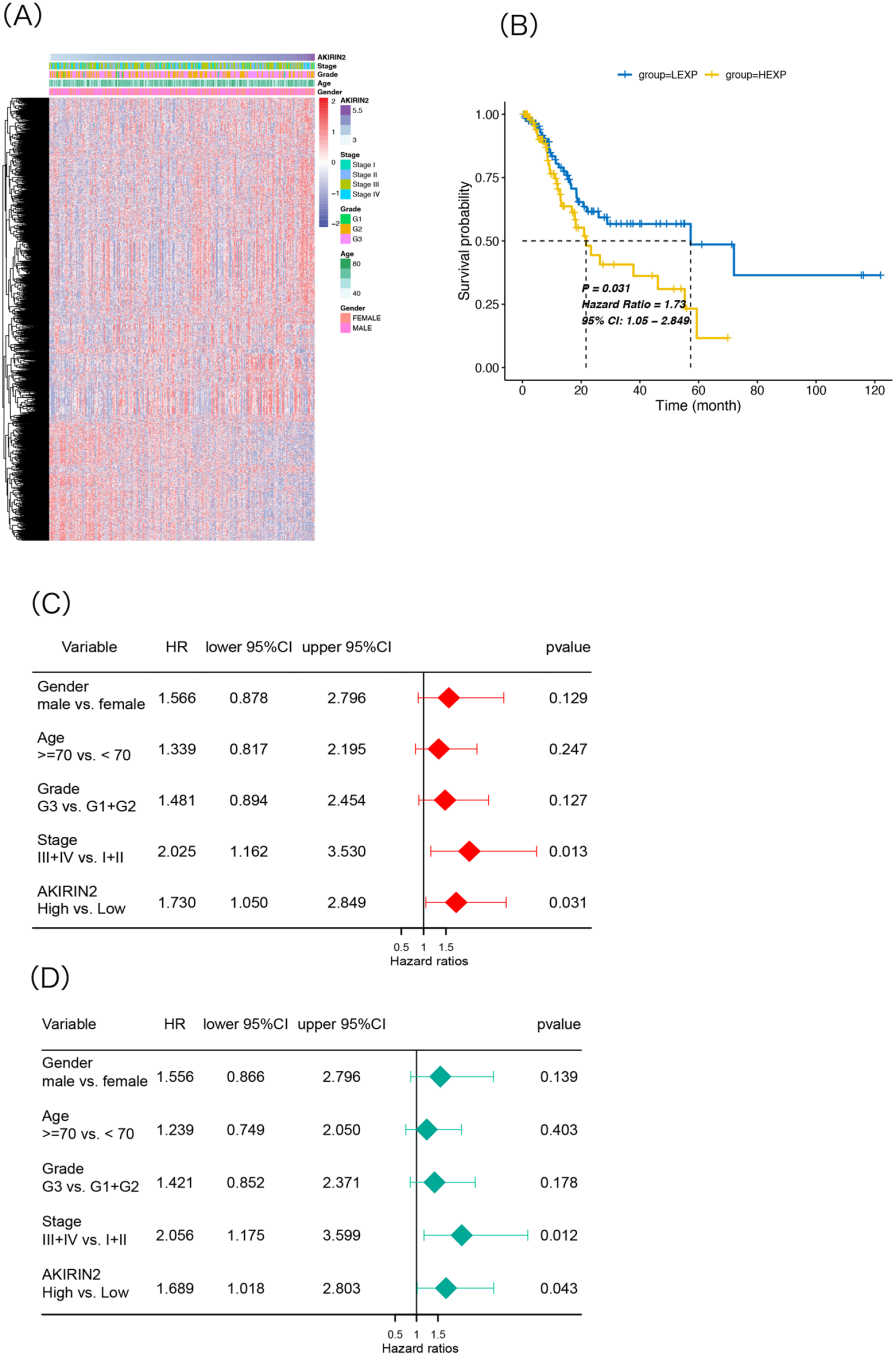
Correlation between AKIRIN2 expression of GA and clinical features in TCGA-STAD. (**A**) Heatmap of DEGs in 277 samples and correlation between AKIRIN2 expression level and different clinical features. (**B**) Survival curve of AKIRIN2 in high- and low-expression groups. (**C**) Univariate Cox regression analysis of the correlation between AKIRIN2 expression and other clinical factors. (**D**) Multivariate Cox regression analysis of the correlation between the expression of AKIRIN2 expression and other clinical factors. All the figures were generated by R software (version 4.0, https://www.r-project.org). (**A**,**B**) Were generated with ‘pheatmap’ and ’ggplot2’ R packages respectively. (**C**,**D**) Were generated by ‘forest’ R package.

Next, we explored the correlation between AKIRIN2 and clinical features. According to the expression of AKIRIN2, patients were divided into high- and low-expression groups. We collated the clinical data of the two groups, including gender, age, tumor grade, and tumor stage, to evaluate the correlation between clinical parameters and AKIRIN2 (Table 1). However, we did not find any significant difference in the clinical features between the two groups. The heatmap showed the correlation between clinical characteristics with AKIRIN2 expression and the clustering of differentially expressed genes in the high- and low-expression AKIRIN2 groups (Fig. 2A). Next, K–M analysis assessed whether the expression of AKIRIN2 has an effect in GA over survival (Fig. 2B). Patients in the low-expression group had a longer survival time than those in the high-expression group (P = 0.031). Then, univariate and multivariate Cox regression analyses were performed to research the correlation between overall survival (OS) and clinical characteristics. The univariate Cox regression forest plot demonstrated that tumor stage III, IV (P = 0.013), and the level of AKIRIN2 (P = 0.031) were identified as independent risk factors to affect the OS of GA patients (Fig. 2C). In addition, multivariate Cox proportional hazards regression analysis determined the clinical features that contributed to the OS. The findings also showed that stage III, IV and AKIRIN2 were significantly connected with OS, as shown in Fig. 2D. These results suggested that AKIRIN2 expression may be an independent clinical factor affecting the OS and prognosis of GA patients.

**Table 1:** Correlation between the expression level of AKIRIN2 and clinical characteristics.

Characteristics	Number of cases (%)	Expression of AKIRIN2		*P* value
		Low (number of cases)	High (number of cases)	
**Gender**				
Female	98 (35.4)	47	51	0.673
Male	179 (64.6)	92	87	
**Age**				
≥ 70	108 (38.9)	49	59	0.201
< 70	169 (61.1)	90	79	
**Tumor grade**				
G1 + G2	109 (39.4)	59	50	0.350
G3	168 (60.6)	80	88	
**Tumor stage**				
Stage1 + 2	122 (44.1)	64	58	0.581
Stage3 + 4	155 (55.9)	75	80

### Enrichment analysis of AKIRIN2-related signaling pathways

**Figure 3:**
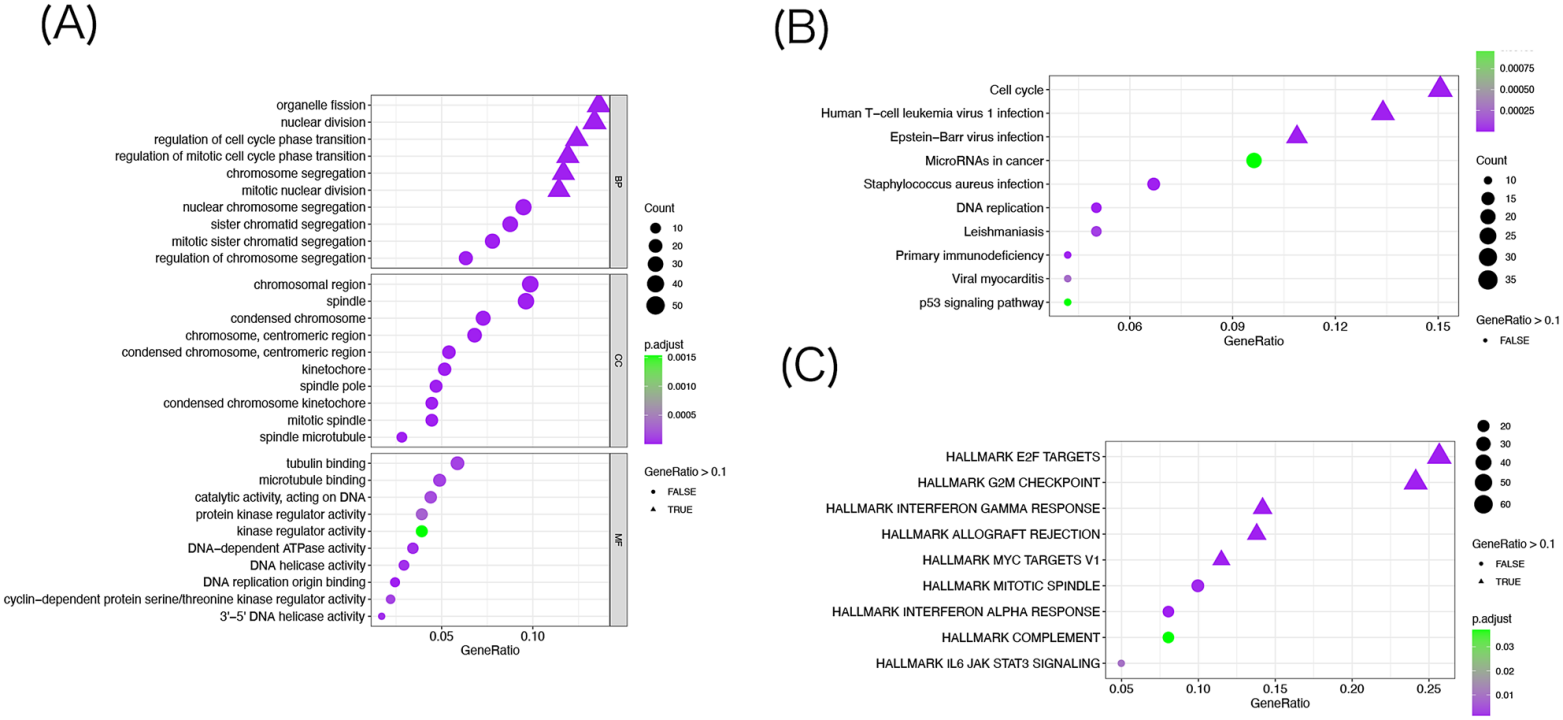
Go (**A**), KEGG (**B**), and HALLMARK (**C**) enrichment analysis was performed for the up-regulated genes in DGEs between the high and low expression groups. KEGG data were provided by Kanehisa Laboratories^20–22^ (https://www.kegg.jp/kegg/). Figures were generated by R software (version 4.0, https://www.r-project.org). (**A**–**C**) Were generated with ‘enrichGO’, ‘cnetplot’ and ‘cluster profiler’ R packages respectively.

In order to explore the functional phenotype between high- and low-expression groups, the GO function and KEGG and Hallmark pathway were analyzed in the upregulated DEGs. The GO annotation results revealed that the biological processes were primarily associated with organelle fission, nuclear division, regulation of cell cycle phase transition, regulation of mitotic cell cycle phase transition, chromosome segregation, and mitotic nuclear division (Fig. 3A). Moreover, the KEGG pathway analysis identified the genes involved in cell cycle, T-cell leukemia virus 1 infection, Epstein–Barr virus infection, microRNAs in cancer, *Staphylococcus aureus* infection, DNA replication, Leishmaniasis, primary immunodeficiency, viral myocarditis, and p53 signaling pathway (Fig. 3B). The bubble chart of Hallmark gene sets showed the genes enriched in E2F targets, G2M checkpoint, gamma response, allograft rejection, myc target v1, mitotic spindle, interferon-alpha response, complement, and IL6-JAK-STAT3 signaling (Fig. 3C). The findings validated that DEGs are mostly enriched in cell cycle progression, proliferation, and differentiation-related pathways, thereby indicating that AKIIN2 plays a major role in regulating cell proliferation and cell survival, which is consistent with previous studies^34^.

### AKIRIN2 expression is correlated with immune infiltration and immune cells

**Figure 4:**
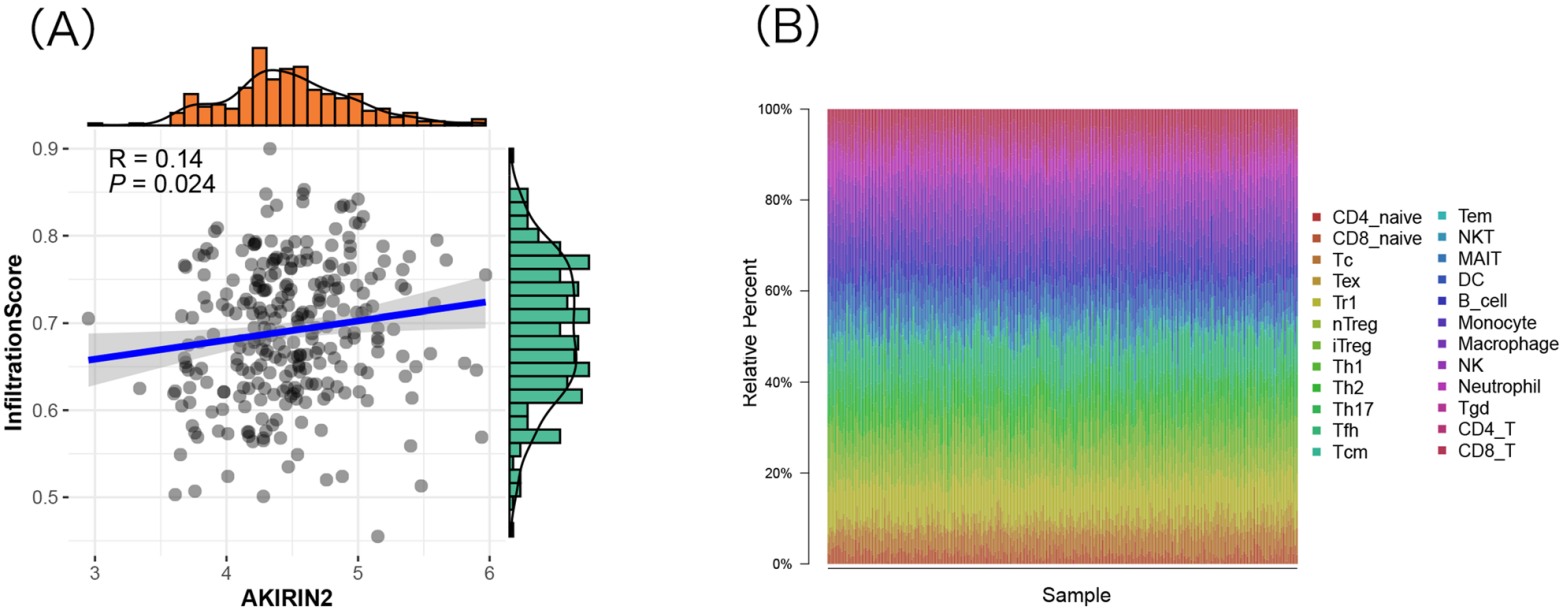

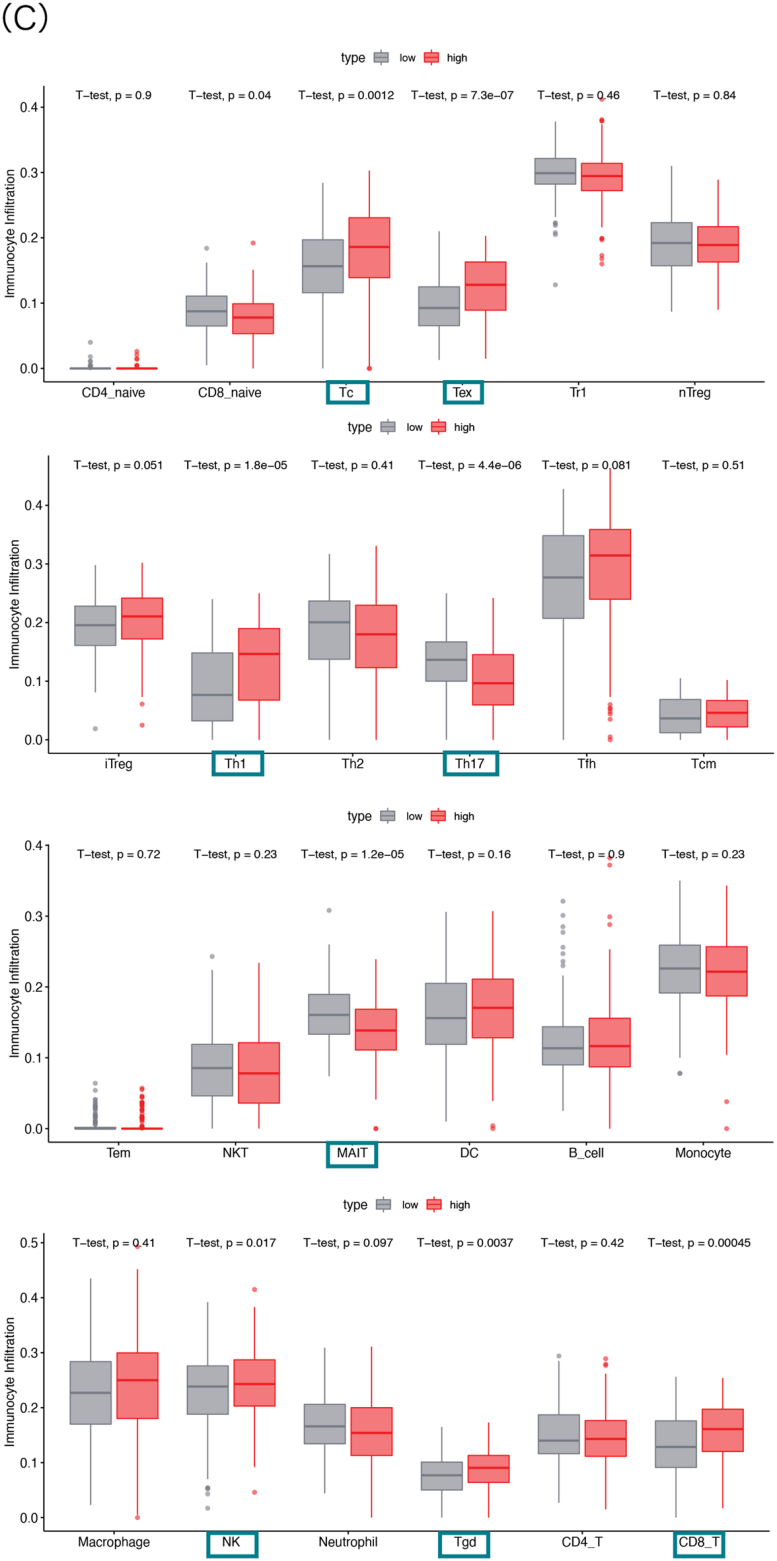
The correlation of AKIRIN2 and immune infiltration in GA. (**A**) The analysis of AKIRIN2 expression level immune infiltration in GA (P value = 0.024 < 0.05, R = 0.14). (**B**) Immune cells by ImmuCellAI based on 277 GA samples. (**C**) Comparison of 24 immune cells in AKIRIN2 high and low expression groups. Figures were generated by R software (version 4.0, https://www.r-project.org). (**A**–**C**) Were both generated with the ‘ggplot2’ R package.

To investigate the effect of AKIRIN2 expression on different immune cell types in the GA microenvironment, we downloaded the distribution of 24 immune cells of GA samples from ImmuCellAI and analyzed the linear correlation between the expression of AKIRIN2 and the expression of infiltration score by Pearson’s correlation analysis (Fig. 4A). The AKIRIN2 expression was significantly correlated with immune scores (R = 0.14, P < 0.024). Figure 4B shows the distribution of 24 types of immune cells in 277 samples. Next, we compared the level of immune cells in the high-and low-expression AKIRIN2 groups. A total of 8 different immune cells were related to the expression of AKIRIN2 (P < 0.05), as shown in Fig. 4C. Tc, Tex, Th1, NK, Tgd and CD8^+^ T were positively correlated with AKIRIN2 expression, while MALT and Th17 were negatively correlated.

### Immune cells closely related to AKIRIN2 expression

**Figure 5:**
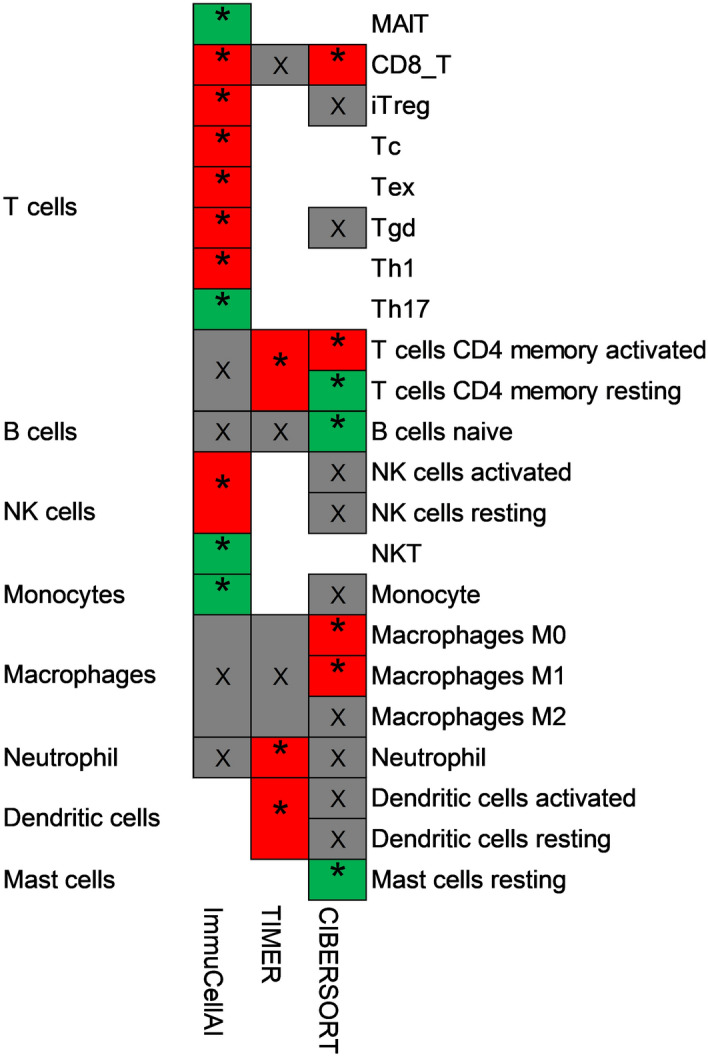
The correlation between AKIRIN2 and immune cells. Three methods (CIBERSORT, TIMER and ImmuCellAI) were used to validate the correlation between AKIRIN2 and immune cells (*P < 0.05, red means up-regulated and green means down-regulated). The figure was generated by Microsoft Office Excel (version 2020, https://www.microsoft.com).

Moreover, we studied the correlation between AKIRIN2 expression and the immune cell abundance based on Pearson’s correlation coefficient analysis in ImmuCellAI (Table 2). We found that the high expression of AKIRIN2 was positively correlated with the expression of Tgd, NK, iTreg, Tc, CD8^+^ T, Th1 and Tex but negatively correlated with the expression of Th17, MALT, NKT, Monocyte (P < 0.001). Next, a combination of CIBERSORT, TIMER, and ImmuCellAI analyzed the correlation between various immune cell subtypes and AKIRIN2 expression to verify that the immune cells in GA are specifically affected by AKIRIN2 expression. Figure 5 shows the expression of immune cells in three methods. All relevant data are within the paper and its supplementary information files. CD8^+^ T cell count is positively correlated with AKIRIN2 in both ImmuCellAI and CIBERSORT. CD4^+^ memory T cells is also activated, which is consistent with the results from TIMER and CIBERSORT.

**Table 2:** Correlation between AKIRIN2 and the abundance of immune cells.

Cell types	Expression of AKIRIN2	
	Pearson's correlation	*P* value
Th17	− 0.323	< 0.001
MAIT	− 0.289	< 0.001
NKT	− 0.169	0.005
Monocyte	− 0.158	0.009
Tgd	0.126	0.036
NK	0.182	0.003
iTreg	0.196	< 0.001
Tc	0.204	< 0.001
CD8_T	0.212	< 0.001
Th1	0.295	< 0.001
Tex	0.316	< 0.001

### Predicting response to chemotherapy and immunotherapy

**Figure 6:**
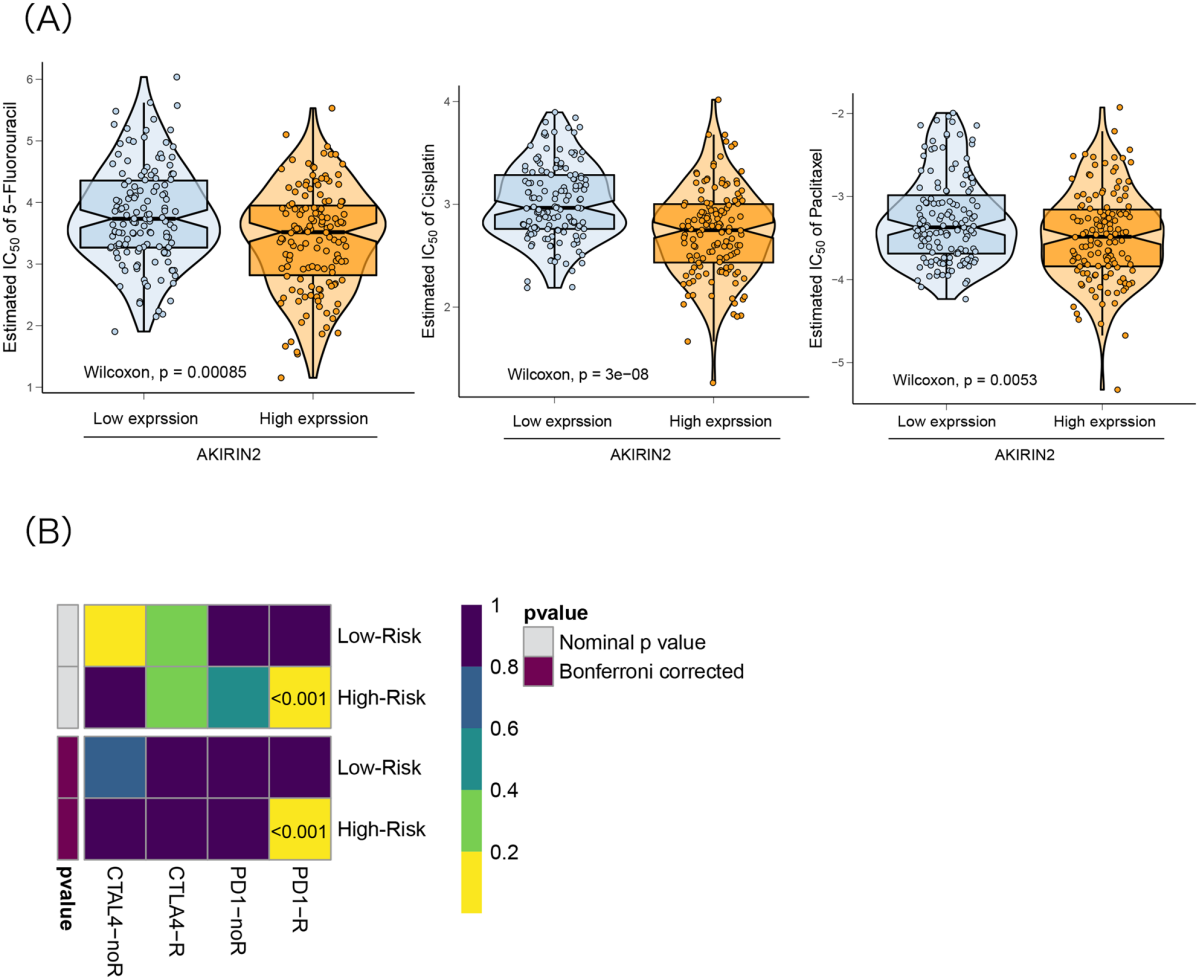
Relationship between AKIRIN2 and chemotherapy/immunotherapy. (**A**) Comparison of chemosensitivity between high and low AKIRIN2 expression groups Reference (P < 0.05). (**B**) Submap analysis manifested that high expression AKIRIN2 could be more sensitive to the immunotherapy (Bonferroni-corrected P < 0.001). Figures were generated by R software (version 4.0, https://cloud.r-project.org). (**A**,**B**) Were generated with the ‘ggplot2’ and ‘pheatmap’ R package respectively.

Herein, we also assessed the role of AKIRIN2 in chemotherapy and immunotherapy for GC. Chemotherapy is a common treatment for patients with advanced GC. The IC50 for each sample in the TCGA-STAD dataset was estimated based on the predictive model of chemotherapeutics. The ridge regression method was used to train the prediction model on the GDSC cell line data set, and the tenfold cross-validation method was used to evaluate the prediction accuracy. Cisplatin, paclitaxel, and 5-fluorouracil showed significant differences in the estimated IC50 against the high-expression group. The results showed that GA with high expression of AKIRIN2 is sensitive to commonly used chemotherapy (Fig. 6A).

The submap algorithm was used to predict the possibility of responding to immunotherapy. Subclass mapping was used to compare the expression profiles of the two groups with the dataset of an open melanoma treatment cohort of 47 melanoma patients receiving programmed cell death protein-1 (PD-1) or cytotoxic T lymphocyte-associated protein-4 (CTLA-4) immunosuppression. Finally, a significant correlation (P < 0.001) was established between the high expression of AKIRIN2 and PD-1 responders (Fig. 6B), suggesting that patients in the high-expression AKIRIN2 group adequately responded to anti-PD-1 therapy.

## Discussion

GC is a global health issue^35^. Although early recognition and treatment are possible, most cases are diagnosed at a late stage, and the majority of the patients with GC diagnosis die of the disease^3^. Gastroscope is critical for diagnosing gastric diseases. Gastroscopy-based screening is utilized for the early detection and treatment of patients with early GC. However, it is intrusive, requires abundant human resources and large population analysis^36^. Also, effective biomarkers are essential. Some previous studies have provided information on GC biomarkers. For example, Zhang et.al identified a panel of specific signatures by dissecting the single-cell transcriptome network for the precise diagnosis of early gastric cancer^37^; hitherto, the biomarkers that can predict the survival of GA patients are not efficient. Early studies identified a strong expression of AKIRIN2 in several tumor cell lines^38^. The same results were obtained in animal experiments of cholangiocarcinoma. Moreover, high AKIRIN2 levels have also been confirmed to be associated with poor CCA prognosis^8^. Next, we explored the possibility of AKIRIN2 as a potential biomarker of GA prognosis and analyzed the interaction between tumor immune infiltration and AKIRIN2 expression in GA. The following K–M analysis also confirmed this finding, and the high-expression AKIRIN2 group of GA showed a poor OS curve. Univariate and multivariate Cox regression analyses assessed the correlation between OS and clinical characteristics and demonstrated that the level of AKIRIN2 and tumor stage were two risk factors affecting the survival of GA patients. In addition, we did not find any significant difference in the clinical features between high- and low-expression AKIRIN2. Therefore, the ability of AKIRIN2 to predict clinical prognosis is independent of the clinical features.

Surgery^39^, chemotherapy^40^, targeted therapy^41^, and immunotherapy^42^ are the main strategies for the treatment of advanced GC. In patients who miss the opportunity of surgical treatment, prolonging survival and improving the quality of life are the primary goals aimed to be achieved via chemotherapeutics, targeted drugs, and immunotherapy^43^. Intriguingly, GA genotype is a crucial prognostic or predictive factor of the clinical outcome of neoadjuvant chemotherapy^44^. Recently, the US Food and Drug Administration (FDA) announced that Navolizumab combined with chemotherapy is the first approved first-line treatment of patients with advanced or metastatic GC. Several studies have demonstrated the important role of AKIRIN2 in innate immunity^45^, B cell activation, and humoral immune responses^46^. In our study, we also explored the role of AKIRIN2 in chemotherapy and immunotherapy and found that the patients with high expression of AKIRIN2 were suitable for PD-1 inhibitor treatment and common chemotherapy drugs. Therefore, for patients with high AKIRIN2, although the prognosis of patients with this subtype is poor, we can use chemotherapy and immunotherapy to alleviate the disease and prolong survival. However, more clinical data are needed to confirm this.

Signaling pathways play a core role, however, increasing evidence suggested that chromatin regulates gene expression in immune cells^47,48^. A review pointed out that AKIRIN2 exerts a distinct role in nuclear factor-κB and chromatin remodelers and is involved in the transcriptional regulation of macrophages and B cells^47^. In the current study, we conducted an enrichment analysis for the upregulated DEGs in the high- and low-expression groups and found that function and pathways are enriched in regulating the cell proliferation and survival in GA. Combined with the correlation between AKIRIN2 and immune cells in the follow-up analysis, we speculated that AKIRIN2 is involved in the regulation of gene expression of immune cells by affecting the chromatin in GA.

The immune cells in the tumor microenvironment lose the anti-tumor function but promote tumor growth^49^. If cytotoxic CD8^+^ T cells enter the tumor, they can destroy the cells. However, the cells and factors of TME provide an inhibitory environment, resulting in the loss of the original function of CD8^+^ T cells^50^, i.e., CD8^+^ T cells in the TME might not exert the tumor-killing effect due to the decline in their function^51^. A new state of persisting yet functionally compromised CD8^+^ T cells was first proposed by Zajac et al.^52^ and Gallimore et al.^53^. Nowadays, these cells are termed exhausted CD8^+^ T cells and are detected in many disorders^54^, such as chronic infections^55^, cancers^56^, and autoimmune disorders^57^. Barber et al. demonstrated that blocking PD-1 reinvigorates the exhausted CD8^+^ T cells in chronic viral infection^58^. Currently, these cells are considered as the targets of immunotherapies, such as PD-1 blockade^59^. In this study, increased AKIRIN2 expression showed a correlation with poor prognosis and increased immune infiltration levels in CD8^+^T cells and activated CD4^+^ memory T cells. Although the correlation values between AKIRIN2 and immune cells is low, the results of this study are credible according to the analysis experience of other scholars^60,61^. We speculate that it is due to multiple factors of immune microenvironment. This suggests that the interpretation of immune microenvironment should be analyzed from multiple perspectives. Therefore, we speculated that CD8^+^ T cells are positively correlated in the high expression AKIRIN2 group, but due to low activity, the prognosis is poor. Interestingly, the high-expression group is more effective in the treatment of PD-1 inhibitors than the low-expression group. Furthermore, CD4^+^ memory T cells constitute a vital component of the TME that affects tumor occurrence and progression. A previous study showed that a high abundance of CD4^+^ memory T cells is associated with improved survival in patients with GC^59^. In addition, we employed three different methods to judge the degree of immune infiltration, and used all three methods to identify and achieve relatively consistent results for greater credibility. However, three methods generated inconsistency to some extent. This may be due to the different principles behind all three calculation methods, the different predicted types and numbers of immune cells and the complexity involving tumor microenvironment.

There is a shortcoming in this study. Some of the results were inferred by bioinformatics methods without experimental verification. In future, we hope that our peers will study this issue with us.

In summary, AKIRIN2 is highly expressed in GA by our bioinformatics analysis and experimental verification. Increased AKIRIN2 expression correlates with poor prognosis and increased immune infiltration levels in CD8^+^ T cells and CD4^+^ memory T cells. Besides, AKIRIN2 may also be a sensitive index of chemotherapy and immunotherapy.

## Supplementary Information

Supplementary Information.

## Data Availability

The datasets analysed during the current study are available in the Gene Expression Omnibus (GEO) repository, GSE19826 and The Cancer Genome Atlas (TCGA), (https://portal.gdc.cancer.gov/repository).

## Abbreviations

GA: Gastric adenocarcinoma
GC: Gastric cancer
PD-L1: Death-ligand 1
GEO: Gene expression omnibus
CTLA4: Cytotoxic T lymphocyte-associated protein 4
TME: Specific tumor microenvironment
STAD: Stomach adenocarcinoma
TCGA: The Cancer Genome Atlas
GDSC: Genomics of Drug Sensitivity in Cancer
DEGs: Differentially expressed genes
KEGG: Kyoto Encyclopedia of Genes and Genomes
GO: Gene Ontology
TIDE: Tumor immune dysfunction and exclusion
OS: Overall survival
IHC: Immunohistochemistry
MALT: Mucosal-associated invariant T
NK: Natural killer cell

## References

[1] Wu D (2019). Serum biomarker panels for the diagnosis of gastric cancer. Cancer Med..

[2] Smyth EC (2020). Gastric cancer. Lancet.

[3] Sung H (2021). Global cancer statistics 2020: GLOBOCAN estimates of incidence and mortality worldwide for 36 cancers in 185 countries. CA Cancer J. Clin..

[4] Song Z (2017). Progress in the treatment of advanced gastric cancer. Tumour Biol..

[5] Bosch PJ (2020). Akirin proteins in development and disease: Critical roles and mechanisms of action. Cell Mol. Life Sci..

[6] Goto A (2008). Akirins are highly conserved nuclear proteins required for NF-kappaB-dependent gene expression in drosophila and mice. Nat. Immunol..

[7] Krossa S (2015). Down regulation of Akirin-2 increases chemosensitivity in human glioblastomas more efficiently than Twist-1. Oncotarget.

[8] Leng K (2019). Akirin2 is modulated by miR-490-3p and facilitates angiogenesis in cholangiocarcinoma through the IL-6/STAT3/VEGFA signaling pathway. Cell Death Dis..

[9] Rojas A (2020). Gastric tumor microenvironment. Adv. Exp. Med. Biol..

[10] Spill F (2016). Impact of the physical microenvironment on tumor progression and metastasis. Curr. Opin. Biotechnol..

[11] Giraldo NA (2019). The clinical role of the TME in solid cancer. Br. J. Cancer.

[12] Guo Y (2017). Multiscale modeling of inflammation-induced tumorigenesis reveals competing oncogenic and oncoprotective roles for inflammation. Cancer Res..

[13] Yamakoshi Y (2020). Immunological potential of tertiary lymphoid structures surrounding the primary tumor in gastric cancer. Int. J. Oncol..

[14] Larkin J, Hodi FS, Wolchok JD (2015). Combined nivolumab and ipilimumab or monotherapy in untreated melanoma. N. Engl. J. Med..

[15] Goldman MJ (2020). Visualizing and interpreting cancer genomics data via the Xena platform. Nat. Biotechnol..

[16] Ginestet C (2011). ggplot2: Elegant graphics for data analysis. J. R. Stat. Soc..

[17] Ritchie ME (2015). limma powers differential expression analyses for RNA-sequencing and microarray studies. Nucleic Acids Res..

[18] Gene Ontology Consortium: Going forward. *Nucleic Acids Res*. **43**(Database issue), D1049–D1056 (2015). 10.1093/nar/gku1179 PMC4383973 25428369

[19] Kanehisa M (2017). KEGG: New perspectives on genomes, pathways, diseases and drugs. Nucleic Acids Res..

[20] Kanehisa M, Goto S (2000). KEGG: Kyoto encyclopedia of genes and genomes. Nucleic Acids Res..

[21] Kanehisa M (2019). Toward understanding the origin and evolution of cellular organisms. Protein Sci..

[22] Kanehisa M (2021). KEGG: Integrating viruses and cellular organisms. Nucleic Acids Res..

[23] Liberzon A (2015). The Molecular Signatures Database (MSigDB) hallmark gene set collection. Cell Syst..

[24] Arneth B (2019). Tumor microenvironment. Medicina (Kaunas).

[25] Miao YR (2020). ImmuCellAI: A unique method for comprehensive T-cell subsets abundance prediction and its application in cancer immunotherapy. Adv. Sci. (Weinh).

[26] Chen B (2018). Profiling tumor infiltrating immune cells with CIBERSORT. Methods Mol. Biol..

[27] Li T (2017). TIMER: A web server for comprehensive analysis of tumor-infiltrating immune cells. Cancer Res..

[28] Yang W (2013). Genomics of Drug Sensitivity in Cancer (GDSC): A resource for therapeutic biomarker discovery in cancer cells. Nucleic Acids Res..

[29] Geeleher P, Cox N, Huang RS (2014). pRRophetic: An R package for prediction of clinical chemotherapeutic response from tumor gene expression levels. PLoS One.

[30] Jiang P (2018). Signatures of T cell dysfunction and exclusion predict cancer immunotherapy response. Nat. Med..

[31] Hoshida Y (2007). Subclass mapping: Identifying common subtypes in independent disease data sets. PLoS One.

[32] Roh W (2017). Integrated molecular analysis of tumor biopsies on sequential CTLA-4 and PD-1 blockade reveals markers of response and resistance. Sci. Transl. Med..

[33] Sideras K (2019). Circulating levels of PD-L1 and Galectin-9 are associated with patient survival in surgically treated Hepatocellular Carcinoma independent of their intra-tumoral expression levels. Sci. Rep..

[34] Murphy LO, Blenis J (2006). MAPK signal specificity: The right place at the right time. Trends Biochem. Sci..

[35] Park JY, Herrero R (2021). Recent progress in gastric cancer prevention. Best Pract. Res. Clin. Gastroenterol..

[36] Horiguchi N (2017). A comparative study of white light endoscopy, chromoendoscopy and magnifying endoscopy with narrow band imaging in the diagnosis of early gastric cancer after *Helicobacter pylori* eradication. J. Gastrointestin. Liver Dis..

[37] Zhang P (2019). Dissecting the single-cell transcriptome network underlying gastric premalignant lesions and early gastric cancer. Cell Rep..

[38] Komiya Y (2008). A novel binding factor of 14-3-3beta functions as a transcriptional repressor and promotes anchorage-independent growth, tumorigenicity, and metastasis. J. Biol. Chem..

[39] He W (2015). Surgical interventions for gastric cancer: A review of systematic reviews. Int. J. Clin. Exp. Med..

[40] Reddavid R (2018). Neoadjuvant chemotherapy for gastric cancer. Is it a must or a fake?. World J. Gastroenterol..

[41] Lazăr DC (2016). New advances in targeted gastric cancer treatment. World J. Gastroenterol..

[42] Kelly RJ (2017). Immunotherapy for esophageal and gastric cancer. Am. Soc. Clin. Oncol. Educ. Book.

[43] Johnston FM, Beckman M (2019). Updates on management of gastric cancer. Curr. Oncol. Rep..

[44] Machlowska J (2020). Gastric cancer: Epidemiology, risk factors, classification, genomic characteristics and treatment strategies. Int. J. Mol. Sci..

[45] Tartey S, Takeuchi O (2015). Chromatin remodeling and transcriptional control in innate immunity: Emergence of Akirin2 as a novel player. Biomolecules.

[46] Tartey S (2015). Essential function for the nuclear protein Akirin2 in B cell activation and humoral immune responses. J. Immunol..

[47] Tartey S, Takeuchi O (2016). Akirin2-mediated transcriptional control by recruiting SWI/SNF complex in B cells. Crit. Rev. Immunol..

[48] Smale ST, Natoli G (2014). Transcriptional control of inflammatory responses. Cold Spring Harb. Perspect. Biol..

[49] Whiteside TL (2008). The tumor microenvironment and its role in promoting tumor growth. Oncogene.

[50] Nouri-Shirazi M (2000). Dendritic cells capture killed tumor cells and present their antigens to elicit tumor-specific immune responses. J. Immunol..

[51] Maimela NR, Liu S, Zhang Y (2019). Fates of CD8+ T cells in tumor microenvironment. Comput. Struct. Biotechnol. J..

[52] Zajac AJ (1998). Viral immune evasion due to persistence of activated T cells without effector function. J. Exp. Med..

[53] Gallimore A (1998). Induction and exhaustion of lymphocytic choriomeningitis virus-specific cytotoxic T lymphocytes visualized using soluble tetrameric major histocompatibility complex class I-peptide complexes. J. Exp. Med..

[54] Ye B (2015). T-cell exhaustion in chronic hepatitis B infection: Current knowledge and clinical significance. Cell Death Dis..

[55] Wherry EJ, Kurachi M (2015). Molecular and cellular insights into T cell exhaustion. Nat. Rev. Immunol..

[56] Huang AC (2017). T-cell invigoration to tumour burden ratio associated with anti-PD-1 response. Nature.

[57] McKinney EF (2015). T-cell exhaustion, co-stimulation and clinical outcome in autoimmunity and infection. Nature.

[58] Barber DL (2006). Restoring function in exhausted CD8 T cells during chronic viral infection. Nature.

[59] Ning ZK (2020). Molecular subtypes and CD4(+) memory T cell-based signature associated with clinical outcomes in gastric cancer. Front. Oncol..

[60] Alcaraz-Sanabria A (2019). Genomic signatures of immune activation predict outcome in advanced stages of Ovarian cancer and basal-like breast tumors. Front. Oncol..

[61] Xiao Z (2020). TGFβ2 is a prognostic-related biomarker and correlated with immune infiltrates in gastric cancer. J. Cell Mol. Med..

